# Metronidazole-Loaded Chitosan Nanoparticles with Antimicrobial Activity Against *Clostridium perfringens*

**DOI:** 10.3390/pharmaceutics17030294

**Published:** 2025-02-24

**Authors:** Anca Niculina Cadinoiu, Delia Mihaela Rata, Oana Maria Daraba, Leonard Ionut Atanase, Cristina Elena Horhogea, Jean-François Chailan, Marcel Popa, Alexandru Carauleanu

**Affiliations:** 1 Academician Ioan Hăulică Research Institute, Faculty of Medicine, Apollonia University of Iași, 700511 Iași, Romania; maria.mary2019@yahoo.com (O.M.D.); leonard.atanase@yahoo.com (L.I.A.); marpopa2001@yahoo.fr (M.P.); 2 Academy of Romanian Scientists, 050045 Bucharest, Romania; 3 Department of Public Health, Faculty of Veterinary Medicine, Ion Ionescu de la Brad Iasi University of Life Sciences, 700489 Iasi, Romania; cristina.horhogea@iuls.ro; 4 Materiaux-Polymeres-Interfaces-Environnement Marin (MAPIEM) Laboratory, University of Sud Toulon-Var, CEDEX, 83957 La Garde, France; chailan@univ-tln.fr; 5 Department of Obstetrics and Gynecology, Grigore T. Popa University of Medicine and Pharmacy, 700111 Iasi, Romania; drcarauleanu@yahoo.com

**Keywords:** nanoparticles, chitosan, tannic acid, metronidazole, antimicrobial activity

## Abstract

**Background/Objectives**: Even with improvements in surgical techniques and the application of appropriate antibiotic prophylaxis, wound infections are still major public health problems in low- and middle-income countries. This study proposes the design of new particulate polymeric matrices based on chitosan (CS) for the controlled release of Metronidazole (MTZ), in order for it to be used for the treatment of *Clostridium perfringens* infections. **Methods**: The nanoparticles were prepared via inverse emulsion using tannic acid (TA) and sodium tripolyphosphate (TPP) as cross-linking agents. The ratio of CS to TPP, the concentration of CS solution, and the ratio of CS to TA were varied to optimize the synthesis procedure. Nanoparticles have been characterized based on several points of view in order to correctly correlate their properties with synthesis parameters. **Results**: The FTIR spectra of the analyzed nanoparticles confirmed both the formation of hydrogen bonds between CS and TA and the ionic cross-linking of CS with TPP. The average diameters of the nanoparticles ranged from 70 to 170 nm, whereas the zeta potential values were around 8 mV. Their swelling degree in a weak basic environment, as well as the drug loading/release capacity was influenced, as expected, by the synthesis parameters. The obtained nanoparticles were tested in vitro to evaluate their behavior in the blood environment, the cytotoxic effect, and the antimicrobial activity of nanoparticles loaded with MTZ against *Clostridium perfringens* cultures. **Conclusions**: The in vitro obtained results demonstrate that these non-hemolytic and non-cytotoxic particles can be efficient drug delivery systems for the treatment of *Clostridium perfringens* in wound infections.

## 1. Introduction

Wound infections are still serious public health problems in low- and middle-income countries, even though there have been many improvements in surgical techniques and the application of appropriate antibiotic prophylaxis [1]. The increased risk of wound infection is mainly due to factors such as age, other adjacent diseases, and patient hygiene before and after surgery, but also post-surgical complications such as wound colonization with bacteria, such as *Clostridium perfringens* [2]. Gas gangrene, also known as clostridial myonecrosis, is a bacterial infection that damages tissues and is most often caused by *Clostridium bacteria* (most commonly, *C. perfringens*) [3]. These bacteria release toxins that destroy blood cells, blood vessels, and muscle tissue causing severe blistering, swelling, and discoloration of the skin [4].

Metronidazole (MTZ) belongs to a class of imidazolytes, and can still be successfully used for the treatment of trichomoniasis, amoebiasis, and giardiasis. Other known advantages of this drug are its low cost, effective activity against pathogenic anaerobic bacteria, favorable pharmacokinetic and pharmacodynamic properties, as well as its minor adverse effects [5]. Moreover, MTZ is a drug that has been used successfully to treat *Clostridium perfringens* infections [1].

Many studies have focused on the development of new dressing materials, which ideally should simulate the extracellular matrix (ECM) to promote cell proliferation and facilitate good antimicrobial properties [6]. Among the different types of nanomaterials, nanoparticles have presented an effective treatment alternative for wound healing due to their unique characteristics of acting as a therapeutic platform and their ability to penetrate the wound area [7]. Until now, MTZ has been loaded into different types of nanoparticles (based on polymers or lipids) [8,9,10].

Nanohydrogels, compared to other systems that encapsulate hydrophobic and hydrophilic compounds, present an advantage because the cross-linked network functions as a matrix that holds the absorbed liquid medium and modulates the diffusion of active principles [11].

Considering the above, the present study proposed the development and evaluation of nanoparticle systems based on CS/TA/TPP for the release of MTZ in order for it to be used in the treatment of *Clostridium perfringens* infections. By replacing the covalent cross-linking agent used in our previous studies [12,13] with tannic acid, a natural cross-linker, it was possible to obtain a non-toxic nanoparticle system that was characterized by using several points of view: structural, morphological, biomaterial properties, and antimicrobial properties against the strain of *Clostridium perfringens*.

Chitosan (CS), one of the most used polymers for obtaining nanohydrogels, has excellent biocompatible and biodegradable properties because it comes from natural sources and the products resulting from its degradation are easily metabolizable [14]. CS [(1, 4)-2-amino-2-deoxy-D-glucan] is deacylated chitin and has excellent biodegradable and biocompatible characteristics, with a unique polymeric cationic character, as well as gel- and film-forming properties [15,16]. It can be used in the development of drug control releasing systems, such as sponges, films, beads, microbeads (microspheres), and nanoparticles [17,18,19]. Degradation products of CS are not toxic, immunogenic, or carcinogenic. To increase the mechanical properties of drug delivery systems based on CS, covalent cross-linking agents are usually used. To overcome the toxicity problems associated with chemical cross-linkers, such as glutaraldehyde, natural alternatives, such as genipin, hydroxycinnamic, citric, ferulic, and tannic acids, have been studied [20,21,22].

Tannic acid (TA) is a polyhydric phenolic compound, a mixed gallotannin composed of high molecular weight hydrolysable polyphenols, including gallic acid esters and glucose. TA is found in the bark of chestnut, oak, hemlock, and in the iron of certain plants. TA has been extensively studied for its antioxidant, anti-inflammatory, anticarcinogenic or antimutagenic properties [23,24]. This phenolic compound has been shown to have inhibitory action against skin, stomach, and lung tumors caused by polycyclic aromatic hydrocarbon carcinogens and N-methyl-N-nitrosourea in mice [25,26]. Owing to the pKa value between 7 and 8, as a function of the degree of dissociation, TA partially becomes hydrolyzed into glucose and gallic acid moieties under mild acidic/basic conditions [25].

The novelty of this study consists of the preparation of efficient drug delivery systems in combining organic chemical compounds of natural origin with antimicrobial properties, such as chitosan and tannic acid, for the development of a biocompatible nanosystem capable of including Metronidazole, which has an enhanced antimicrobial activity against *Clostridium perfringens*, by minimizing his dermatological side effects.

Metronidazole-loaded Chitosan nanoparticles were obtained, by varying the concentration of chitosan solutions, CS/TA weight ratio and CS/TPP weight ratio, and their physicochemical, biological and antimicrobial properties were assessed by different methods, such as: FT-IR, SEM, UV-Vis spectroscopy, MTT assay.

## 2. Materials and Methods

### 2.1. Materials

Low molecular weight chitosan (CS) (90% deacetylation degree), tannic acid (TA), Tween80, Span80, sodium tripolyphosphate (TPP), hexane, and acetone were purchased from the Sigma Aldrich, St. Louis, MO, USA. Toluene was obtained from the Honeywell Specialty Chemicals Seelze GmbH, Seelze, Germany. Metronidazole 99% (MTZ) was purchased from Alfa Aeser, Karlsruhe, Germany. Cryopreserved primary human dermal fibroblast (HDF) cells and reagents used for cytotoxicity tests were purchased from Thermo Fisher Scientific, Waltham, MA, USA. The fresh bacterial culture of *Clostridium perfringens* (ATCC 13124) was obtained from Sanimed, Giurgiu, Romania.

### 2.2. Preparation Method

Until now, many methods have been used to synthesize CS nanoparticles, taking into account their size, stability, and toxicity. To be easily reproducible on an industrial scale, the methods must also be of low difficulty, with as few preparation and purification steps as possible. The main objective of the present study was to design such nanoparticles, with low toxicity, for drug loading/release with potential applications in infectious pathologies. To achieve this goal, a hydrophilic model antibiotic, MTZ, was investigated. To obtain the nanoparticles, tannic acid (TA) and sodium tripolyphosphate (TPP) were used as cross-linking agents in different ratios and concentrations. The ability of TA to form multiple hydrogen bonds together with CS, and the successful obtaining of three-dimensional networks has been previously reported in the literature [25,26]. In this paper, several parameters that could influence the characteristics of the obtained systems were analyzed, such as the CS/TA molar ratio, the concentration of the CS solution, and the ratio between CS and TPP. The parameters were expected to influence both the physicochemical characteristics and the drug loading/release capacity of the obtained nanoparticles.

**Figure 1:**
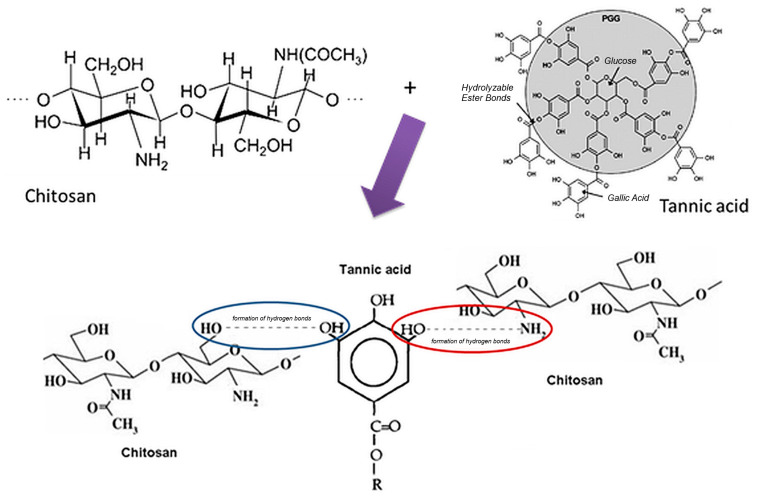
Proposed mechanism for the formation of hydrogen bonds between CS and TA.

No chemical reactions, such as ionic cross-linking, occur between TA and CS. However, all the components of the system have hydrogen atoms bound to electronegative elements (O and N), which leads to the hypothesis of the formation of numerous hydrogen bonds between the two compounds, thus ensuring the stability of the network. Taking into account the above, the following mechanism for the formation of hydrogen bonds between CS and TA (Figure 1) can be proposed.

Chitosan-based nanoparticles (PCTAs) were obtained by inverse emulsion using tannic acid and sodium tripolyphosphate as crosslinkers. An experiment program was developed considering three variables: CS/TPP weight ratio (*w*/*w*), concentration of chitosan solutions (%) (*w*/*v*), and CS/TA weight ratio (*w*/*w*) (Table 1).

These parameters were chosen based on a previously published paper where CS/TA hydrogels films were investigated [27].

CS-based nanoparticles were obtained following several essential steps, as follows. The required amounts of CS for each PCTA sample were dissolved in 40 mL of diluted acetic acid solution (1%, *v*/*v*). The 10 mL solution of TA was placed in distilled water at different concentration, and was added to the CS solution under stirring after the complete dissolution of CS. After stirring for 30 min, 1 g of Tween80 was solved into the aqueous solution and the formed mixture was added drop-wise into 200 mL toluene containing 4 g of Span80 to form a W/O emulsion under stirring at 6000 rpm, using an UltraTurrax homogenizer (IKA T 25 digital ULTRA-TURRAX, Staufen, Germany); therefore, a 4:1 weight ratio of Span80 and Tween80 was used for all experiments.

The 5 mL TPP solution was added drop by drop into the obtained emulsion and the stirring was continued for another 30 min. Finally, the nanoparticles were separated by centrifugation at 8000 rpm for 30 min and the oil phase was removed. The sediment that remained was washed several times with acetone, distilled water, acetone and finally with hexane to extract the non-reacted reagents and the two surfactants. After washing with hexane, the obtained PCTA nanoparticles were allowed to dry at 37 °C in the oven.

### 2.3. Nanoparticles Characterization

#### 2.3.1. FTIR Analysis

The structural characteristics of the obtained nanoparticles were determined through FTIR spectroscopy using a single reflection ATR accessory QATR-S of a Shimadzu IRSpirit spectrometer, (Schimadzu Europa GmbH, Duisburg, Germany). Spectra, in absorbance mode, were recorded at room temperature in the range 4000–400 cm^−1^ at a resolution of 4 cm^−1^.

#### 2.3.2. Morphology of Nanoparticles

The mean diameter of the PCTA nanoparticles and the size distribution were evaluated via the laser light diffractometry technique using a Zetasizer Nano ZS from Malvern Panalytical Limited, Worcestershire, UK. All measurements were performed on suspensions of nanoparticles in acetone (2 mL, 0.5% *w*/*v*) after thorough sonication, in order to maintain the particles in an unswollen state, similar to powder. For the calculation of the standard deviation, five successive measurements whose quality reports were good were taken into account.

The surface charge of the nanoparticles obtained and, thus, the stability was measured via laser Doppler micro-electrophoresis at 25 °C after adding them to simulated body fluid (SBF), pH 7.25. The SBF was prepared using the method presented by Marques et al. [28]. Simulated body fluid was chosen because it has ion concentrations nearly equal to those of human plasma. The concentration of PCTAs suspension was 0.01% (*w*/*v*) by diluting it with SBF. The suspension was kept for 10 min in an ultrasonic bath before the analysis. The sample was analyzed in triplicate.

To evaluate the shape, surface and size of the PCTA nanoparticles, a Zeiss Supra 40VP scanning electron microscope (Oberkochen, Germany) was used at the necessary magnification after gold metallization.

#### 2.3.3. Swelling Behavior

The swelling capacity of PCTA nanoparticles was evaluated in SBF by using the gravimetric method [29]. Experiments were performed in triplicate. Known amounts of dry sample (W_0_) were suspended in SBF and the obtained suspension was maintained under gentle stirring (50 rpm) at 37 °C. After 24 h, the nanoparticle suspension was centrifuged and the supernatant was carefully removed. The swollen sample (Ws) were weighed and the degree of swelling (Q%) was calculated according to the following equation:

(1) $$Q\%=\frac{W_{s}-W_{0}}{W_{0}}\times 100$$

#### 2.3.4. Drug Loading and In Vitro Release of Metronidazole

The 50 mg of PCTA nanoparticles were suspended in the 5 mL MTZ (10 mg/mL) solution and dispersed in a sonication bath for 30 min. The suspension was maintained under stirring for 24 h and then the drug loaded particles were separated by centrifugation (10,000 rpm, 15 min) (Hettich Centrifuge, Tuttlingen, Germany). In the next step, these particles were freeze-dried in order to obtain a white powder which was stored at room temperature until further analyses [12,13]. The amount of MTZ loaded in nanoparticles was calculated using an indirect method by determining the MTZ content in the supernatant according to the standard calibration curve by using UV-Vis spectroscopy (Nanodrop One UV–Vis Spectrophotometer, ThermoFisher Scientific, Madison, WI, USA) at 320 nm wavelength. All experiments were performed in triplicate and the entrapment efficiency (E_e_%) was calculated as a ratio between the amount of MTZ loaded into nanoparticles (w_e_MTZ) and the amount of MTZ from the initial solution (w_0_MTZ), according to the following equation:

(2) $$E_{e}\%=\frac{w_{e}MTZ}{w_{0}MTZ}\times 100$$

In vitro release studies were performed using a dissolution apparatus (708-DS Dissolution Apparatus from Agilent Technologies LDA, Bayan Penang, Malaysia) equipped with a sampling station (Sampling Station, 850-DS). The release medium was also SBF (pH = 7.25). For this method, the release medium must be a good solvent for the loaded drug (MTZ) and non-solvent for the obtained nanoparticles. In total, 50 mg of drug-loaded PCTA-M nanoparticles with 1 mL of SBF were placed in dialysis tubes (12,000 Da), sealed well, and then immersed in 200 mL of SBF. A shaking of 50 rpm and a temperature of 37 °C were maintained throughout the experiment. The amount of MTZ released was determined using a Nanodrop One UV–Vis Spectrophotometer. All experiments were performed in triplicate.

The drug release efficiency (R_ef_%) was calculated as a ratio between the cumulative amount of MTZ released at the predetermined times (w_t_MTZ) and the amount of MTZ loaded into nanoparticles (w_e_MTZ) (see Equation (3)).

(3) Ref%=wtMTZweMTZ×100

#### 2.3.5. In Vitro Hemolysis Tests

The hemolysis test is one of the in vitro tests recommended for biomaterials that interact with blood. This interaction may lead to changes in blood properties. For this reason, the influence of PCTA nanoparticles on red blood cells was tested using the spectrophotometric method [30]. The human blood samples used were freshly obtained from healthy non-smoking volunteers after proper informed consent and institutional ethical approval. In total, 5 mL of collected blood was centrifuged at 2000 rpm for 5 min to sediment the red blood cells (RBCs). Plasma was removed, and RBCs were washed several times with saline (0.9%) from Hemofarm, Vršac, Serbia. A saline solution was finally added over the RBC to obtain 25 mL of suspension. In total, 0.5 mL of the RBC suspension was put in contact with 0.5 mL of the PCTA particle suspension to obtain different concentrations (100 µg nanoparticles/mL, 50 µg nanoparticles/mL, and 25 µg nanoparticles/mL). Positive control (100% lysis) and negative control (0% lysis) samples were also prepared. RBC suspensions, together with PCTA nanoparticles, were incubated at 37 °C for 90 and 180 min. To prevent the sedimentation of RBCs and nanoparticles, the samples were shaken every 30 min. After the incubation time expired, the samples were centrifuged at 3000 rpm for 5 min, and the supernatant remained at rest for another 30 min at room temperature. The absorbance of oxyhemoglobin released into the supernatant was measured spectrophotometrically at 540 nm.

#### 2.3.6. Cytotoxicity Tests

The in vitro cytotoxic effects of PCTA-2, PCTA-3, and PCTA-6 samples were determined on the human dermal fibroblast cell line. For this assay, HDF cells were cultured in DMEM supplemented with 10% FBS, an antibiotic cocktail consisting of 1% (*v*/*v*) penicillin–streptomycin and 1% (*v*/*v*) non-essential amino acids. The culture medium was changed every other day. Cells were incubated at 37 °C in a humidified atmosphere of 5% CO_2_ in the air, using an MCO-5AC-PE incubator, Panasonic Healthcare, Sakata, Japan. Cells were allowed to grow to 80% confluence in culture flasks (75 cm^2^) and then trypsinized with a 0.25% trypsin solution at 37 °C for 3 min. A fresh medium at room temperature was added to neutralize the trypsin. Cells were then separated by centrifugation (ROTOFIX-32A centrifuge, Hettich, Tuttlingen, Germany) and resuspended in the fresh medium. Viable cells were cultured in 96-well plates, and PCTA nanoparticles were added to obtain different concentrations (30, 90, and 150 µg of PCTA nanoparticles/mL medium). Fibroblast viability was assessed after 24 h and 48 h of incubation in culture media conditioned with PCTA nanoparticles. After treatment, the cells were processed according to the cell viability assay with MTT [31], and absorbance was measured at 570 nm using a Multiskan FC automated microplate reader (Thermo Fisher Scientific Instruments, Shanghai, China) and Skanlt Software 4.1.

#### 2.3.7. The Antimicrobial Activity

The susceptibility of the bacterial strain of *Clostridium perfringens* to the tested samples was determined by using the diffusion method according to the guidelines provided by the Clinical and Laboratory Standards Institute (CLSI) [32,33].

The test sample was represented by PCTA3-M, in different concentrations: PCTA3-M-1 (1.76 mg of nanoparticles loaded with MTZ/mL), PCTA3-M-2 (1.06 mg of nanoparticles loaded with MTZ/mL), PCTA3-M-3 (0.70 mg of MTZ-loaded nanoparticles/mL) and the corresponding controls PCTA3-1, PCTA3-2 and PCTA3-3. A fresh bacterial culture of *Clostridium perfringens* (ATCC 13124), obtained in a blood agar after 24 h incubation at 37 °C and anaerobiosis (GENbag anaer bioMérieux) was used for these tests. In a sterile Petri dish, 1 mL of the 0.5 McFarland microbial suspension (1.5 × 10^8^ CFU—colony forming unit) was dispersed in saline and the molten Brucella media base (Oxoid) was added and thoroughly homogenized. After the agar solidified, 6 wells (holes with a diameter of approximately 10 mm each) were cut and filled with 50 μL of the tested samples. The plates were incubated for 48 h at 37 °C under an anaerobic atmosphere.

The interpretation of the results was based on the ability of substances with antimicrobial action to inhibit the multiplication of microorganisms, translated by the appearance of a clear zone of different sizes around the wells. The diameter of the zone of inhibition was measured with a ruler and expressed in millimeters (mm). The experiment was performed in triplicate. Mean values were calculated using an online standard deviation calculator [34].

Statistical analysis. The way in which the parameters that were varied and their interactions influenced particle size, PDI, zeta potential, swelling degree, loading efficiency, n value, hemolysis degree, and cell viability was evaluated by a three-way ANOVA [31], where each of them was considered the dependent variable. For particle size, PDI, zeta potential, swelling degree, loading efficiency, and the n value of the following independent variables were considered: the ratio CS/TPP; the concentration of CS solutions, and the CS/TA ratio. For the hemolysis degree and cell viability, the independent variables considered were as follows: sample type, concentration, and time. For the interpretation of the ANOVA results, the *p*-values obtained were taken into account (see Tables S1, S2, and S4). The *p*-values less than 0.05 indicate that the variables or interactions between variables have a statistically significant effect on the dependent variables. The three-way ANOVA analysis was carried out using Mathematica (version 14, Wolfram Research, Champaign, IL, USA).

The statistical significance of the results obtained from antimicrobial tests was analyzed by using the one-way ANOVA with the Tukey post hoc test. The experimental results with *p*-values < 0.05 were assumed to be statistically significant [35].

## 3. Results and Discussion

### 3.1. FTIR Analysis

**Figure 2:**
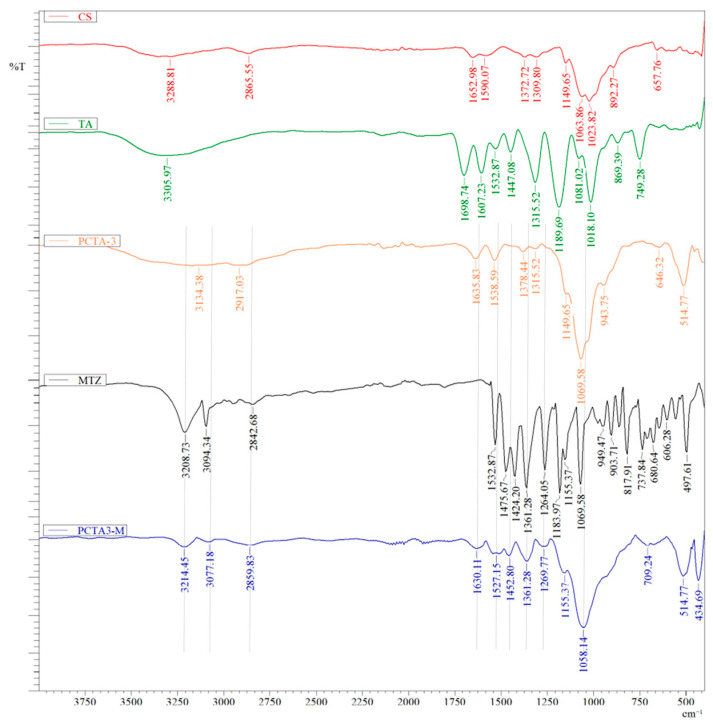
FTIR spectra of CS, TA, PCTA-3 sample, free MTZ and MTZ-loaded PCTA3-M sample.

FTIR spectroscopy highlighted the structural characteristics of the new systems obtained through the transmission technique. For this analysis, samples with the same ratio between CS and TPP, but with different concentrations of the chitosan solution, respectively, with the highest ratio between CS/TA (sample PCTA-3) and the lowest ratio (sample PCTA-7) were discussed, as these two samples have a more obvious influence of TA as compared to other samples. FTIR spectra of the initial components of the system CS and TA, typical drug-free nanoparticles sample, free MTZ, and MTZ-loaded PCTA3 sample are provided in Figure 2.

The spectrum of pure CS (Figure 2) shows peaks around 892 and 1163 cm^−1^ (the saccharide structure), peaks at 1652 and 1309 cm^−1^ (amides I and III), and peaks at 1372 cm^−1^ (the CH_3_ symmetrical deformation). Peaks at 1063 cm^−1^ (the C–O stretching vibration) and the broad peak at approximately 3288 cm^−1^ (the amine N–H symmetrical vibration) are also visible.

The spectrum of TA displays a strong band centered at 3305 cm^−1^, assigned to the hydroxyl (OH) groups’ stretching vibrations due to the wide variety of hydrogen bonding between OH groups. The deformation vibration of the carbon–carbon bonds in the phenolic groups absorbs in the region of 1500–1400 cm^−1^, whereas that is around 1447 cm^−1^, which is the stretching vibrations of C-C aromatic groups. Also, it can be observed that TA contains some aromatic esters due to the signal characteristic bands of the carbonyl groups: C=O stretching vibration at 1698 cm^−1^ and C-O at 1100–1300 cm^−1^. The sharp peak at 749 cm^−1^ corresponds to the distortion vibration of C=C in benzene rings. All these peaks are similar with those presented in the literature [36].

Analyzing the FTIR spectrum of typical PCTA3 sample, the following can be noted: (i) the band around 1635 cm^−1^, assigned to antisymmetric deformation N-H vibrations in NH^3+^ ion and around 1069 cm^−1^, corresponding to symmetric and antisymmetric stretching vibrations in PO_3_ group, which confirms the formation of ionic cross-links between NH^3+^ groups of CS and TPP ions [37]; (ii) the band around 1149 cm^−1^, assigned to aromatic C-O bond stretching and at 1538 cm^−1^ for the aromatic C=C bond. The presence of these two peaks demonstrates the reaction between CS and TA.

The FTIR spectrum of free MTZ shows the characteristic peak of OH stretching at 3208 cm^−1^, at 3094 cm^−1^ corresponding to C-H aromatic stretching whereas the peaks at 1532 cm^−1^ were assigned to C=C (imidazole ring). Moreover, vibrational peaks at 1475 and 1424 cm^−1^ can be attributed to CH_2_ bending and C-C stretching, respectively, while peaks at 1361 cm^−1^ were assigned to N=O asymmetric stretching. Furthermore, absorption band at 1275–1069 cm^−1^ were assigned to C-O stretching and C-N stretching, respectively, [38].

In the FTIR spectrum of drug-loaded PCTA3-M sample are observed, first, the characteristic peak of MTZ at 1361 cm^−1^ which is a proof of the drug loading inside the particles or drug adsorption at the surface of the particles. Moreover, it can be noticed that some characteristic peaks of both free MTZ and drug-free nanoparticles are shifted which is indicative of the hydrogen interactions between the polymer matrix and MTZ. For example, peaks at 1058, 1269, 1452, 1527, 1630, 3077 and 3214 cm^−1^ are either down or upfield shifted as compared with those from the spectra of free MTZ and PCTA3 sample.

### 3.2. Morphological Characteristics

The size of drug delivery systems is an important physicochemical characteristic because it can influence cellular absorption, pharmacokinetics, tissue distribution or clearance [39]. In order to determine the size of the nanoparticles in a state as close as possible to the unswollen one, the measurements were performed in suspension in acetone. The values of the average diameter and the polydispersity index (PDI) are presented in (Table 2).

**Figure 3:**
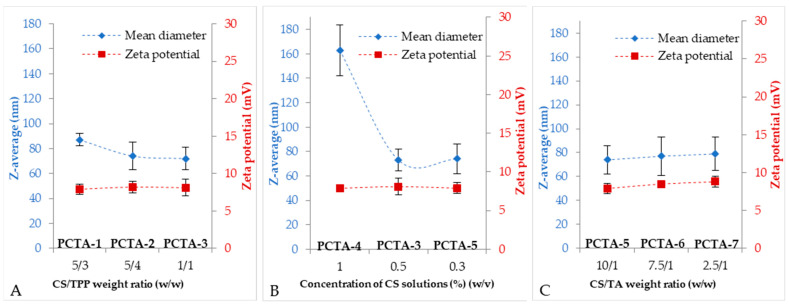
Mean diameters and zeta potential values plotted against the varied parameters.

The size of the nanoparticles is influenced, as expected, by the preparation parameters. When the ratio between CS and the ionic cross-linker decreases (↘ CS/TPP), a decrease in the size of the nanoparticles is observed (Figure 3A). The amount of CS was kept constant, and the amount of ionic cross-linkers added was changed. When the ratio decreases, the amount of cross-linkers actually increases and, thus, this increases the degree of cross-linking, with the formed network being tighter, thus reducing the size of the formed particles.

For the samples where the concentration of the CS solution was varied (Figure 3B), an influence was also observed; with the increase in the concentration of CS, the size of the particles also increased. The biggest difference was visible when the concentration increased from 0.5% (PCTA-3) where a size of approximately 74 nm was obtained to 1% (PCTA-4) and where the size was approximately 163 nm (Table 2).

When the ratio between CS and TA was changed (Figure 3C) (the amount of TA was kept constant, and the amount of CS changed). It was observed that with its decrease, the particle size slightly increased.

As illustrated in Figure S1 (Supplementary File) for two typical samples, the size distribution is monomodal even if PDI values ranged between 0.32 and 0.59. This index gives us information about the size distribution of the nanoparticles; samples that have a wider range of particle sizes have PDI values greater than 0.7, while samples containing equal-sized particles have PDI values less than 0.2 [39]. When CS-based nanoparticles are characterized, these values are often encountered since CS is a polymer known for its mucoadhesive properties [40], which lead to the formation of aggregates in the case of particulate systems [41].

The agglomeration tendency of the nanoparticles is also highlighted by the zeta potential values (Table 2 and Figure 2), which are around 8 mV. In the literature, the nanoparticles obtained by cross-linking CS with TPP have been shown to have a positive surface charge, with values between 14 and 71 mV, depending, in general, on the obtained parameters [29]. In the basic medium, the amine groups not involved in cross-linking are not quaternized so that no electrostatic repulsions occur that can determine the increased values of the zeta potential.

Statistical results regarding the influence of parameters on particle size, PDI and zeta potential are provided in Table S1 (Supplementary File). As can be seen from the *p*-value analysis, the variation in chitosan concentration is a significant factor regarding particle size and the CS/TA ratio has a significant influence on zeta potential values.

**Figure 4:**
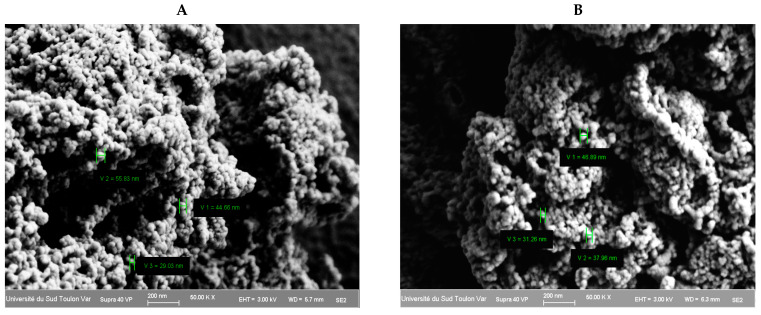
SEM images of samples PCTA-3 (**A**) and PCTA-7 (**B**).

Figure 4 shows images obtained using scanning electron microscopy (SEM) that reveal the morphology of the PCTA-3 and PCTA-7 synthesized nanoparticles. For SEM analysis, the same samples that were chosen for FTIR analysis were discussed. As can be seen, the morphology of the nanoparticles obtained was relatively homogeneous, with a quite constant particle size distribution, with a spherical shape and a smooth surface.

The size of the nanoparticles, as can be seen in the SEM images, varied from 30 to 55 nm, with no noticeable differences between the two analyzed samples.

### 3.3. Swelling Studies

The drug loading and release capacity of polymer nanoparticles depends on their behavior in the aqueous environment, more precisely on their ability to swell and allow drug molecules to penetrate through the polymer network. To determine the swelling capacity of the obtained nanoparticles, a buffer solution was used that has ion concentrations nearly equal to those of human plasma (simulated body fluid—SBF with pH 7.25) [28].

**Figure 5:**
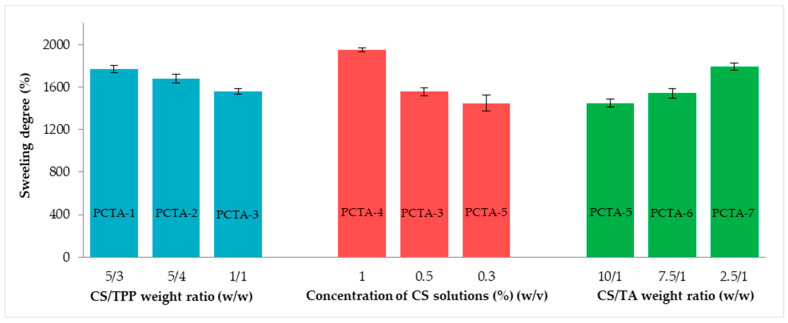
The swelling degree of PCTA particles after 24 h in simulated biological fluid. Data are presented as mean ± SD, n = 3.

The swelling degree after 24 h was quantified by the gravimetric method as described in a previous study [30]. The maximum swelling degree was also influenced by the parameters that were varied during the synthesis, as can be seen in Figure 5.

At constant CS and TA concentrations, the decrease in the CS/TPP ratio, which corresponds to an increase in the TPP amount, leads to a decrease in the degree of swelling. This behavior can be attributed to the increase in the cross-linking degree, owing to an increased number of NH_3_ groups from CS, which are involved in the cross-linking reaction with the phosphate group of TPP, leading to the formation of inter- and intramolecular bonds. This increased cross-linking degree reduces the flexibility of CS chains and, implicitly, diminishes the amount of the buffer solution that can penetrate into the meshes of the network due to a reduction in the available volume inside these meshes. Moreover, the concentration of the polymer solution used in the synthesis has a notable influence on the degree of swelling; as was the case for the particle size, an increase in the concentration of CS, at constant TPP and TA concentrations, lead to an increase in the degree of swelling. Furthermore, the swelling properties also depend on the amount of tannic acid used for the preparation of particles. Changing the ratio between CS and TA produced an obvious variation in the swelling degree values. In a weakly basic environment, tannic acid forms phenolate ions and thus, by raising the amount of tannic acid, the inherent electrostatic repulsions from the network also increase, which lead to an enhanced swelling degree. In addition, the hydrophobicity of the system is improved due to the presence of numerous phenolic hydroxyl groups in TA which have a positive influence on the water uptake [40].

Statistical results regarding the influence of parameters on swelling degree are provided in Table S2 (Supplementary File); ns: nonsignificant.

### 3.4. Drug Loading and In Vitro Release of Metronidazole

It is clear that loading the drug during synthesis could be the easiest way to facilitate encapsulation, but since MTZ is a hydrophilic molecule, post-synthesis loading was preferred because the drug can be eliminated in a large amount during the purification steps, as this involves multiple washes with water and acetone, both of which are solvents of MTZ. In fact, we observed this phenomenon during some preliminary tests. Taking into account the above, MTZ loading was accomplished via diffusion, with nanoparticles being swollen in a drug solution for 24 h. Table S3 (Supplementary File) reveals the colloidal characteristics of drug-loaded nanoparticles and Table 3 shows the amount of the loaded drug and entrapment efficiency after centrifugation and drying were performed via lyophilization. As noticed in Table S1, an important increase was observed for the particles sizes which are in the range of 412 to 690 nm. The intensity size distribution curves for MTZ-loaded particles are presented in Figure S2 (Supplementary File). The PDI values are lower compared to the empty particles, indicating a monomodal size distribution. These lower values can be due to the supplementary purification step. Moreover, the zeta potential values are slightly lower compared with drug-free particles.

The MTZ were loaded via diffusion, and the entrapment efficiency was influenced by the swelling degree of the nanoparticles. Thus, in accordance with the results obtained for swelling, when the ratio between CS and the ionic cross-linking agent decreased, there was a decrease in the amount of drugs loaded in the nanoparticles due to an increase in the cross-linking density and, therefore, a decrease in the swelling degree. The highest amount of MTZ was loaded in the PCTA4-M sample (0.20 mg/mg particles) for which the highest CS concentration was used. Moreover, this sample is characterized by the highest swelling capacity, which means that a higher volume of drug aqueous solution can diffuse inside the network meshes. For the other two samples (PCTA3-M and PCTA5-M), for which the polymer concentration was varied, loading efficiencies were obtained close in value and lower than the one obtained in the case of the PCTA4-M sample (Table 3). A slight increase in loading efficiency was observed when the ratio between CS and TA decreased. This behavior may be due to the presence of the higher number of hydroxyl TA groups, which allow for the formation of hydrogen bonds not only with water but also with MTZ.

The statistical results regarding the influence of parameters on entrapment efficiency are provided in Table S2 (Supplementary File); ns: nonsignificant

**Figure 6:**
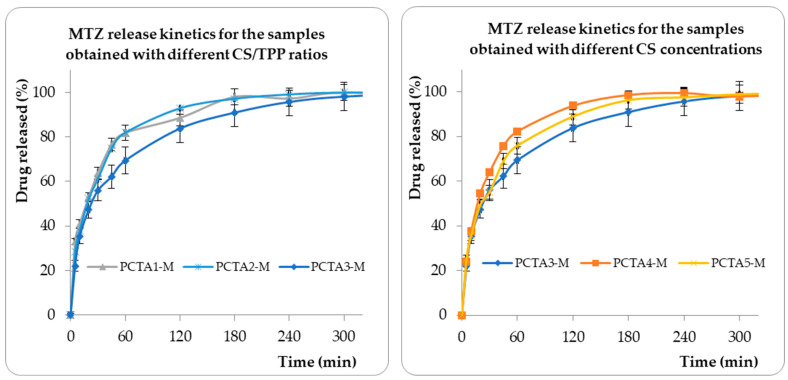
MTZ release profiles in simulated body fluid (SBF) at pH 7.25 and 37 °C. Data are presented as mean ± SD, n = 3.

The drug release tests were performed in vitro, using a dissolution system equipped with an autosampler station. In fact, 50 mg of drug-loaded PCTA-M nanoparticles were suspended in 1 mL of SBF and placed in dialysis tubes that were immersed in 200 mL of SBF. The released kinetics (Figure 6) were followed in the simulated body fluid (pH 7.25), maintaining constant temperature (37 °C) and light stirring speed (50 rpm) to simulate conditions close to those in human organisms. The release profile, in identical conditions, of 10 mg of free MTZ is provided in Figure S3 in the Supplementary File.

As can be seen from Figure 6, the release of MTZ occurred in the following two stages: (i) in the first 10–20 min, there was a release of approximately 40% of the loaded drug that can be attributed to the MTZ adsorbed on the surface of the nanoparticles; (ii) in the second stage, there was a slow increase until equilibrium in drug release that can be attributed to the diffusion of MTZ. Compared to the release profile of free MTZ, provided in Figure S3, a controlled and sustained drug release from the nanoparticles can be noticed.

Information on the mechanism of drug transport and release from particles was obtained using the Ritger–Peppas kinetic model [41] and the obtained data are provided in Table 4.

The n values, which are almost all higher than 0.4, as shown in Table 4, indicate that a Fickian diffusion mechanism describes the release of drugs from these spherical particles. The release rate constant (k) values depend on the physical and structural properties of both the drug and the polymeric matrix. The k values from Table 2 indicate that the drug rate release is the highest for the PCTA1-M sample, whereas the lowest values are obtained for PCTA3-M and PCTA4-M samples. Moreover, the higher values of R2 are clear proof that these results fit perfectly the Ritger–Peppas kinetic model.

The statistical results regarding the influence of parameters on the n values are provided in Table S2 (Supplementary File); ns: nonsignificant.

### 3.5. In Vitro Hemolysis Tests

PCTA samples were tested in vitro to evaluate their behavior in the blood environment. Different amounts of nanoparticles were added over the suspension of red blood cells with a final concentration of 100 µg nanoparticles/mL, 50 µg nanoparticles/mL, and 25 µg nanoparticles/mL, and the degree of hemolysis was determined at predetermined times (90 and 180 min) by using the spectrophotometric method. In the case of hemolysis, the intermediary ratios for each studied parameter were taken into account. The PCTA-2 sample, with an intermediary CS/TPP ratio, was chosen from the series where the CS/TPP ratio varied while the other two parameters were kept constant. The PCTA-3 sample, with an intermediary CS concentration, was selected from the series where the concentration of CS solutions varied while the other two parameters were kept constant. The PCTA-6 sample, with an intermediary CS/TA ratio, was chosen from the series where the CS/TA ratio varied, while the other two parameters were kept constant. In this way, an overview of all the samples is possible.

**Figure 7:**
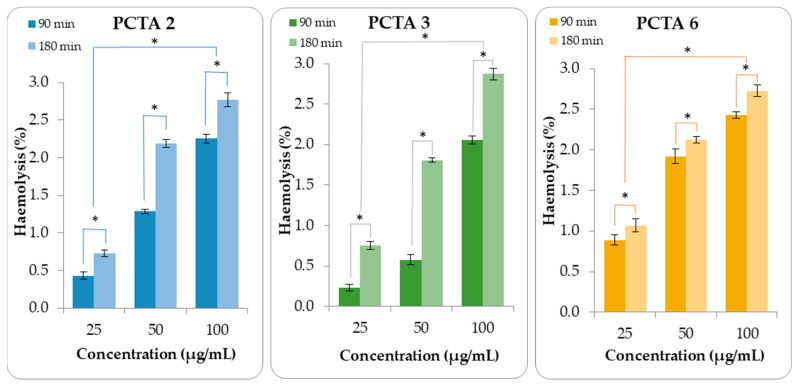
Hemolysis degree after 90 and 180 min of exposure to PCTA samples. Data are presented as mean ± SD, n = 3. Variables that have a statistically significant effect on the hemolysis degree are marked (* *p* < 0.05).

The results obtained from this test (Figure 7) suggest that PCTA nanoparticles show good compatibility with the blood environment because they led to a hemolysis degree lower than 3.5% [42]. From Figure 7, it can also be concluded that the hemolytic percentage increased with the increase in the concentration of nanoparticles.

The statistical results regarding the influence of variables on the hemolysis degree are provided in Tables S3 (Supplementary File). As can be seen from the *p*-value analysis, the sample concentration has a very strong impact on the hemolysis degree. The exposure time to the tested samples also influences hemolysis significantly, while the sample type and all interactions between variables are not significant.

### 3.6. Cytotoxicity Tests

**Figure 8:**
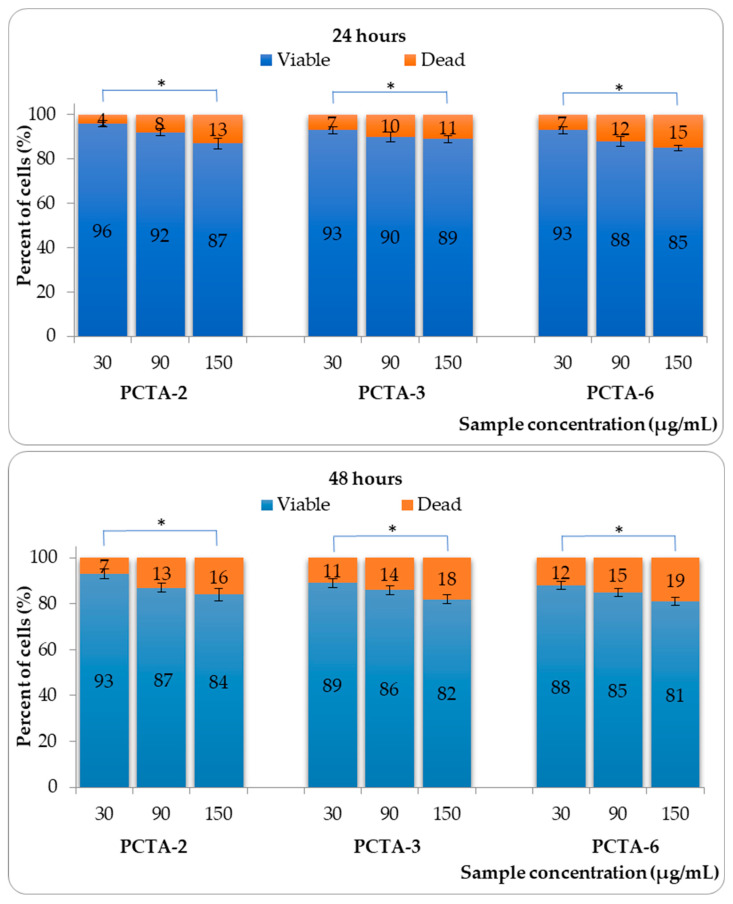
Viability of fibroblast cells in the presence of PCTA-2, PCTA-3, and PCTA-6 samples. Data are presented as mean ± SD, n = 3. Variables that have a statistically significant effect on the viability are marked (* *p* < 0.05).

Human dermal fibroblast (HDF) cells were used as model cells to evaluate the in vitro cytotoxicity of PCTA nanoparticles. The effect of the tested samples on the viability of fibroblasts was evaluated for an incubation period of 24 and 48 h. The results obtained are presented in Figure 8. For cell viability tests, intermediate ratios were taken into account for each parameter studied, as in the case of hemolysis tests.

The nanoparticle samples analyzed induced a non-cytotoxic effect on fibroblast cells after 24 h and 48 h, for all tested concentrations (30, 90, and 150 µg/mL) [43]. It can also be seen that cell viability showed a concentration-dependent effect on the tested cells, increasing the concentration leads to a decrease in viability.

The statistical results regarding the influence of variables on the in vitro cytotoxicity are provided in Table S4 (Supplementary File). The sample type, the concentration of the analyzed samples and the exposure time are very significant variables for cell viability, from a statistical point of view.

### 3.7. The Antimicrobial Activity

**Figure 9:**
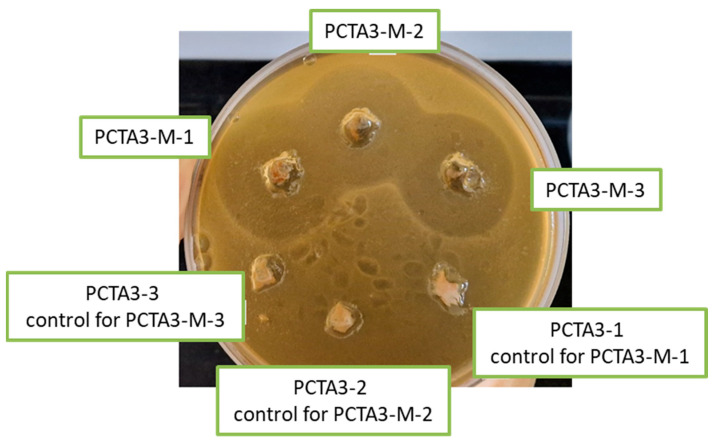
Diffusion susceptibility test against *Clostridium perfringens*.

For the antimicrobial test, the PCTA-3 sample loaded with MTZ (PCTA3-M) was chosen due to optimal values in terms of structure, dimension, morphology, swelling, and release degree. Antibacterial efficacy was evaluated for three different concentrations of the PCTA3-M sample (PCTA3-M-1 (1.76 mg of MTZ-loaded nanoparticles/mL), PCTA3-M-2 (1.06 mg of MTZ-loaded nanoparticles/mL), PCTA3-M-3 (0.70 mg of MTZ-loaded nanoparticles/mL) and the corresponding controls (PCTA3-1, PCTA3-2, and PCTA3-3, nanoparticles without MTZ) (Figure 9).

The diameters of the inhibition zones are provided in Table 5.

According to the data provided in Table 5, the controls (PCTA3-1, PCTA3-2, PCTA3-3) showed a lack of antimicrobial action, demonstrating that the inhibition of bacterial growth was due to the release of loaded MTZ from the polymer matrix. Moreover, it can be observed that by increasing the concentration of loaded MTZ, the inhibition zone increases slightly from 30 to 32 mm. Furthermore, the loaded MTZ has a higher antimicrobial activity than the free drug at equal concentration. Although no reference limits have been established for this bacterial species by the diffusion method, according to EUCAST standards, *Clostridium perfringens* susceptibility for MTZ at a concentration of 5 μg is translated in inhibition zones between 17 and 28 mm [44]. The statistical results provided in Tables S5 (Supplementary File) for the analysis of variance concerning the inhibition diameters against *Clostridium perfringens* between the groups in Table 5 show that the value obtained for the inhibition zone of PCTA3-M-1 sample is the highest and significantly different from other two samples, PCTA3-M-2 and PCTA3-M-3 (*p* < 0.05). However, there are no statistical differences between PCTA3-M-2 and PCTA3-M-3 (*p* > 0.05).

MTZ is a first-line drug for the treatment of human infections caused by anaerobic and microaerophilic bacteria and protozoa. *Clostridium perfringens* is a Gram-positive, anaerobic, spore-forming, and toxin producing bacteria. The mechanism of MTZ action is quite simple and is based on the inhibition of the protein synthesis due to helical DNA structure alteration. MTZ remains in an inactivated form until the up-take by passive diffusion in the cytoplasma of the bacteria cells. The efficacy of this compound in anaerobic bacteria is related to pyruvate ferredoxin/flavodoxin oxidoreductase, an enzyme responsible for a reduction process that produces toxic metabolites. Because this enzyme is active in the anaerobic metabolism, the native resistance of the aerobic bacteria can be explained [45]. Although most *Clostridium perfringens* isolates are susceptible to MTZ, a number of strains with decreased susceptibility have been isolated and identified in clinical and environmental surveys [46,47].

Some studies show that bacteria resistance to MTZ is associated with increased oxygen concentrations in the environment, which favors the selection of aerotolerant anaerobic strains [48]. Other studies mention reduced uptake of the drug for different reasons, increased removal of the active substance from the bacterial cell, or a reduced rate of MTZ activation inside anaerobes [49].

Except the active antimicrobial molecules, the matrix can also influence the positive effect and avoid unwanted side effects. From this point of view, a study from 2017 demonstrated the inhibitory effect of CS against *C. perfringens* spore germination and vegetative growth [50]. TA also can improve antioxidant ability and intestinal health in chickens with necrotic enteritis produced by *C. perfringens* [51,52].

## 4. Conclusions

Nanoparticles based on chitosan and tannic acid were prepared via inverse emulsion using TPP as an ionic cross-linking agent and were loaded with MTZ for use in the treatment of *Clostridium perfringens* infections. To obtain the particles, an experimental plan was used in which the following three parameters were varied: the ratio between CS and TPP, the concentration of the CS solution, and the ratio between CS and TA. The nanoparticles were characterized from several points of view, and it was observed that the degree of swelling and the drug loading/release capacity were influenced by the synthesis conditions. The FTIR spectra of the analyzed nanoparticles confirmed both the formation of hydrogen bonds between CS and TA and the ionic cross-linking of CS with TPP. The average diameters of these spherical nanoparticles ranged from 70 to 170 nm for empty particles, whereas the loading of MTZ led to an important increase in the average size; however, this occurred with a slight decrease in the PDI values. The behavior of the nanoparticles in the blood environment was evaluated in vitro, and it was revealed that they are hemocompatible, producing a degree of hemolysis lower than 3.5% for all tested concentrations. The viability of fibroblast cells was determined by using an MTT assay after incubation with nanoparticles for 24 h or 48 h. The obtained results highlight the fact that nanoparticles do not produce toxic effects on fibroblast cells, at all concentrations tested. Antimicrobial tests against *Clostridium perfringens* cultures showed that nanoparticles loaded with MTZ inhibited bacterial growth even at the minimum concentration used (0.70 mg of MTZ-loaded nanoparticles/mL). In order to demonstrate the effective efficiency of these drug delivery systems for the treatment of infected wounds, these positive in vitro results will be further completed with more detailed in vivo tests.

## Figures and Tables

**Table 1:** The parameters that were varied in the preparation of Chitosan-based nanoparticles (PCTA).

Samples	CS/TPP (*w*/*w*)	Concentration of Chitosan Solutions (%) (*w*/*v*)	CS/TA (*w*/*w*)
PCTA-1	5/3	0.5	10/1
PCTA-2	5/4	0.5	10/1
PCTA-3	1/1	0.5	10/1
PCTA-4	1/1	1	10/1
PCTA-5	1/1	0.3	10/1
PCTA-6	1/1	0.3	7.5/1
PCTA-7	1/1	0.3	2.5/1

**Table 2:** Mean diameters and zeta potential values of the prepared nanoparticles suspended in acetone at a concentration of 0.5% *w*/*v* and 25 °C.

Sample	Z-Average ± SD * (nm)	PDI ± SD *	Zeta Potential ± SD * (mV)
PCTA-1	87 ± 5	0.59 ± 0.09	7.9 ± 0.7
PCTA-2	74 ± 11	0.51 ± 0.05	8.2 ± 0.8
PCTA-3	73 ± 9 *	0.55 ± 0.06	8.1 ± 1.1
PCTA-4	163 ± 21 *	0.41 ± 0.03	7.9 ± 0.4
PCTA-5	74 ± 12 *	0.48 ± 0.02	7.9 ± 0.7 *
PCTA-6	77 ± 16	0.40 ± 0.02	8.5 ± 0.3 *
PCTA-7	79 ± 14	0.32 ± 0.01	8.8 ± 0.7 *

* Data presented as mean ± SD; n = 3 (where SD is the standard deviation and n is the number of values). The parameters that have a statistically significant effect on the dependent variable are marked, which in this case are size, PDI, and zeta potential (* *p* < 0.05).

**Table 3:** The amount of MTZ loaded in PCTA nanoparticles and entrapment efficiency. Data are presented as mean ± SD, n = 3.

Samples	mg MTZ Loaded/mg Nanoparticles	Entrapment Efficiency (E_e_%)
PCTA1-M	0.18 ± 0.03	21.4 ± 2.5
PCTA2-M	0.17 ± 0.02	20.2 ± 5.8
PCTA3-M	0.15 ± 0.02	17.1 ± 3.6
PCTA4-M	0.20 ± 0.02	25.2 ± 3.2
PCTA5-M	0.15 ± 0.02	17.9 ± 3.2
PCTA6-M	0.15 ± 0.03	18.2 ± 4.7
PCTA7-M	0.17 ± 0.02	21.1 ± 3.7

**Table 4:** Basic kinetic parameters of the process of drug kinetics release from particles using the Ritger–Peppas kinetic model.

Sample	n	k × 10^2^	R^2^
PCTA1-M	0.38	17.6	0.995
PCTA2-M	0.43	14.1	0.999
PCTA3-M	0.45	11.8	0.975
PCTA4-M	0.50	11.5	0.986
PCTA5-M	0.44	12.7	0.996
PCTA6-M	0.45	12.6	0.989
PCTA7-M	0.41	11.9	0.991

**Table 5:** Inhibition diameters against *Clostridium perfringens*. The significantly different pairs of treatments are marked (* *p* < 0.05).

Samples	Loaded MTZ (µg)	*Clostridium perfringens* (mm)
Free MTZ (4.5 µg)	-	26.8 ± 0.2
PCTA3-1(Control)	0	0
PCTA3-M-1	11.5	32.3 ± 0.3 *
PCTA3-2 (Control)	0	0
PCTA3-M-2	7	30.3 ± 0.3
PCTA3-3 (Control)	0	0
PCTA3-M-3	4.5	29.7 ± 0.3

## Data Availability

The original contributions presented in this study are included in the article/supplementary material. Further inquiries can be directed to the corresponding author.

## Supplementary Materials

The following supporting information can be downloaded at: https://www.mdpi.com/article/10.3390/pharmaceutics17030294/s1, Figure S1. Intensity size distribution curves for PCTA-3 and PCTA-7 samples; Figure S2: Intensity size distribution curves for MTZ-loaded particles; Figure S3. Release kinetics of free MTZ; Table S1. The significance of the results in Table 2 following the ANOVA analysis; Table S2. The significance of the results in Figure 4, Table 3 and Table 4 following the ANOVA analysis; Table S3: Colloidal characteristics of MTZ-loaded particles; Table S4. The significance of the results in Figure 6 and Figure 7 following the ANOVA analysis; Table S5. Summary of the output of the ANOVA analysis of variance concerning the Inhibition diameters against *Clostridium perfringens* among the groups in Table 5.
