# Supplementary material for: Metronidazole-Loaded Chitosan Nanoparticles with Antimicrobial Activity Against Clostridium perfringens
Source: Pharmaceutics. 2025 Feb 24;17(3):294. doi: 10.3390/pharmaceutics17030294 (PMC11944883; doi:10.3390/pharmaceutics17030294)
Supplement: Supplementary file 1 [file pharmaceutics-17-00294-s001.zip › pharmaceutics-3408930-supplementary.pdf]

# Supporting Information

## Contents

Intensity size distribution curves for PCTA-3 and PCTA-7 samples…………………………..…..Figure S1

The significance of the results in Table 2 following the ANOVA analysis……………………... Table S1

The significance of the results in Figure 4, Table 3 and Table 4 following the ANOVA analysis.. Table S2

Colloidal characteristics of MTZ-loaded particles……………………………………..………...….Table S3

Intensity size distribution curves for MTZ-loaded particles………………………………………Figure S2

Release kinetics of free MTZ……………………………………………………………………….Figure S3

The significance of the results in Figure 6 and Figure 7 following the ANOVA analysis………… Table S4

Summary of the output of the analysis of variance concerning the Inhibition diameters against *Clostridium perfringens* among the groups in Table 5 ……………………. …………………………………………Table S5

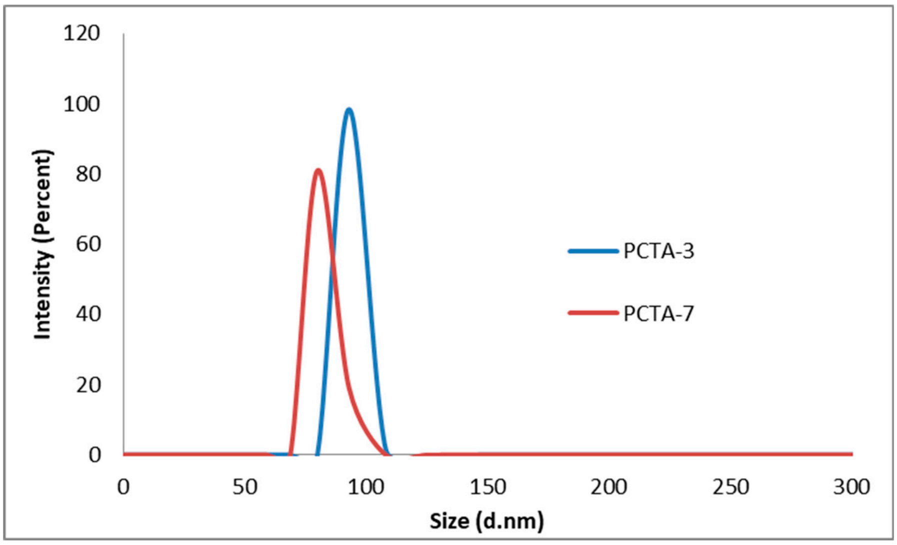


**Figure S1**. Intensity size distribution curves for PCTA-3 and PCTA-7 samples

**Table S1**. The significance of the results in Table 2 following the ANOVA analysis

| Variables | P-value | | |
|---|---|---|---|
| | Concerning the Mean diameters | Concerning the PDI | Concerning the Zeta potential |
| **CS/TPP** | 0.575766 | 0.226525 | 0.189139 |
| **Conc. of CS** | 0.023282 | 0.789782 | 0.0736007 |
| **CS/TA** | 0.279244 | 0.0548503 | 0.0221495 |
| **Conc. of CS and CS/TPP** | 1. | 1.-2.36771x10$^{-8}$*i* | 1.-1.99061x10$^{-8}$*i* |
| **CS/TA and CS/TPP** | 1.-1.59427x10$^{-8}$ *i* | 1. | 1.-1.36038x10$^{-8}$*i* |
| **Conc. of CS and CS/TA** | 1. | 1.-5.91944x10$^{-8}$*i* | 1.-9.3655x10$^{-8}$*i* |

**Table S2**. The significance of the results in Figure 4, Table 3 and Table 4 following the ANOVA analysis

| Variables | P-value | | |
|---|---|---|---|
| | Concerning the swelling degree | Concerning the Entrapment efficiency | Concerning the n values |
| **CS/TPP** | 0.584945 | 0.357941 | 0.073319 |
| **Conc. of CS** | 0.0726795 | 0.074018 | 0.0518885 |
| **CS/TA** | 0.145657 | 0.395563 | 0.18992 |
| **Conc. of CS and CS/TPP** | 1. | 1. | 1. |
| **CS/TA and CS/TPP** | 1. | 1. | 1. |
| **Conc. of CS and CS/TA** | 1. | 1. | 1. |

**Table S3**: Colloidal characteristics of MTZ-loaded particles.

| Sample | Z-average (nm) | PDI | Zeta potential (mV) |
|---|---|---|---|
| **PCTA1-M** | 458 ± 10 | 0.42 ± 0.02 | 4.8 ± 0.5 |
| **PCTA2-M** | 434 ± 9 | 0.36 ± 0.05 | 4.2 ± 0.6 |
| **PCTA3-M** | 512 ± 15 | 0.42 ± 0.04 | 5.1 ± 0.6 |
| **PCTA4-M** | 580 ± 18 | 0.32 ± 0.04 | 4.5 ± 0.1 |
| **PCTA5-M** | 690 ± 20 | 0.38 ± 0.06 | 3.8 ± 0.7 |
| **PCTA6-M** | 591 ± 6 | 0.35 ± 0.02 | 4.5 ± 0.2 |
| **PCTA7-M** | 568 ± 7 | 0.33 ± 0.05 | 3.7 ± 0.3 |

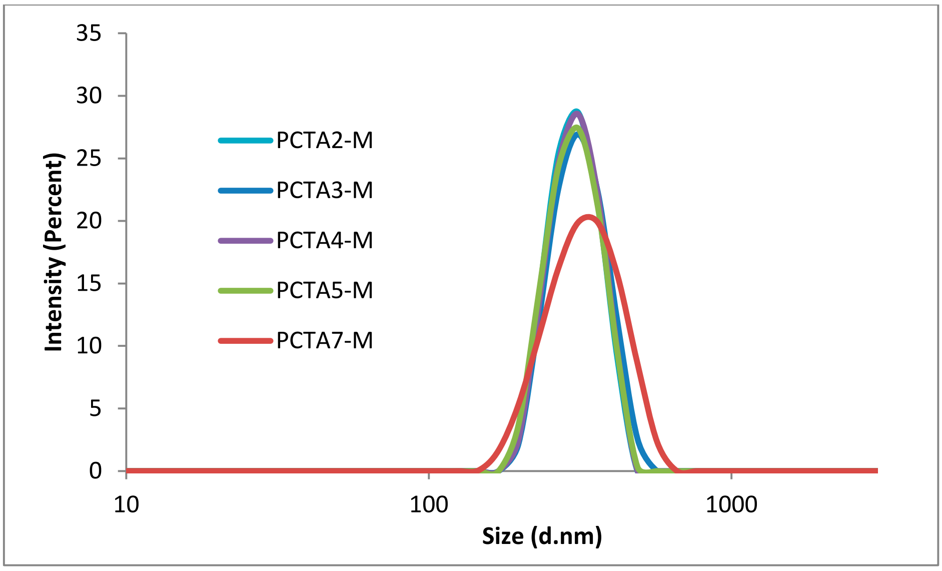


**Figure S2**: Intensity size distribution curves for MTZ-loaded particles

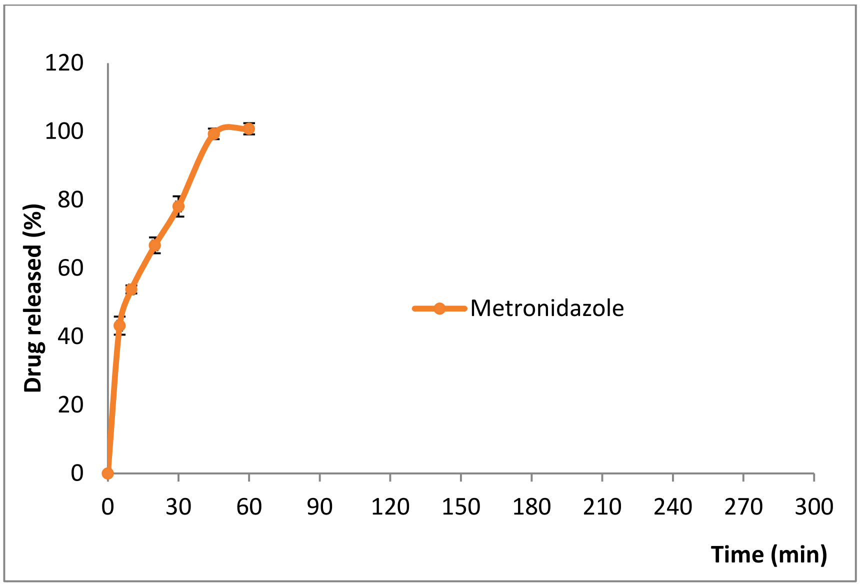


**Figure S3**. Release kinetics of free MTZ

**Table S4**. The significance of the results in Figure 6 and Figure 7 following the ANOVA analysis

| **Variables** | **P-value** | |
|---|---|---|
| | **Concerning the Hemolysis degree** | **Concerning the Viability of fibroblast cells** |
| **Sample type** | 0.311908 | 0.0005007 |
| **Concentration (µg/ml)** | 0.000018 | 3.7349x10$^{-7}$ |
| **Time (min)** | 0.0165148 | 9.04803x10$^{-6}$ |
| **Concentration and Sample type** | 0.574321 | 0.351995 |
| **Time and Sample type** | 0.477237 | 0.795879 |
| **Concentration and Time** | 0.783705 | 0.606764 |
| **Concentration, Time and Sample type** | 0.988187 | 0.751588 |

**Table S5**. Summary of the output of the ANOVA analysis of variance concerning the Inhibition diameters against *Clostridium perfringens* among the groups in Table 5

| **Source** | | **DF** | **Sum of Square** | **Mean Square** | **F Statistic** | **P-value** |
|---|---|---|---|---|---|---|
| Groups (between groups) | PCTA3-M-1 – PCTA3-M-2 – PCTA3-M-3 | 2 | 11.5556 | 5.7778 | 17.3333 | 0.003212 |
| Error (within groups) | | 6 | 2 | 0.3333 | | |
| Total | | 8 | 13.5555 | 1.6944 | | |
| Groups (between groups) | PCTA3-M-1 – PCTA3-M-2 | 1 | 6 | 6 | 18 | 0.01324 |
| Error (within groups) | | 4 | 1.3333 | 0.3333 | | |
| Total | | 5 | 7.3333 | 1.4667 | | |
| Groups (between groups) | PCTA3-M-1 – PCTA3-M-3 | 1 | 10.6667 | 10.6667 | 32 | 0.004813 |
| Error (within groups) | | 4 | 1.3333 | 0.3333 | | |
| Total | | 5 | 12 | 2.4 | | |
| Groups (between groups) | PCTA3-M-2 – PCTA3-M-2 | 1 | 0.6667 | 0.6667 | 2 | 0.2302 |
| Error (within groups) | | 4 | 1.3333 | 0.3333 | | |
| Total | | 5 | 2 | 0.4 | | |
